# Antioxidant activity and total phenolic compounds of *Commiphora gileadensis* extracts obtained by ultrasonic‐assisted extraction, with monitoring antiaging and cytotoxicity activities

**DOI:** 10.1002/fsn3.3339

**Published:** 2023-04-05

**Authors:** Hani Ahmed, Marwan M. A. Rashed, Marwan Almoiliqy, Mohammed Abdalla, Mohanad Bashari, Mohamed Y. Zaky, Zhu Hailin, Taha A. A. Naji, Ahmed Eibaid, Jinpeng Wang, Li‐Ping Jiang

**Affiliations:** ^1^ School of Pharmaceutical Science Nanchang University Nanchang 330006 Jiangxi China; ^2^ School of Biological and Food Engineering Suzhou University Suzhou 234000 Anhui China; ^3^ Department of Medicine and Health Science, College of Medicine and Health Science University of Science and Technology Aden Yemen; ^4^ Department of Translational Molecular Pathology The University of Texas MD Anderson Cancer Center Houston Texas 77030 USA; ^5^ Department of Food Processing, Faculty of Engineering University of El Imam El Mahadi Kosti White Nile 209 Sudan; ^6^ Department of Food Science and Human Nutrition College of Applied and Health Sciences, A'Sharqiyah University Ibra Oman; ^7^ Molecular Physiology Division, Faculty of Science Beni‐Suef University Egypt; ^8^ UPMC Hillman Cancer Center, Division of Hematology and Oncology, Department of Medicine University of Pittsburgh Pittsburgh Pennsylvania 15213 USA; ^9^ Department of Food Science and Technology, Faculty of Engineering and Technology University of Gezira Wad Madani Sudan; ^10^ School of Food and Health Beijing Technology and Business University Beijing China

**Keywords:** anti‐aging, *Commiphora gileadensis*, extraction, HepG2 cells, hydrodistillation, ultrasonic assisted

## Abstract

*Commiphora gileadensis* (*C. gileadensis*) has been identified and linked with various health benefits and pharmaceutical potential for its phytochemical activities and chemical constituents. This study aimed to evaluate ultrasonic‐assisted extraction (USE) technique for total phenols content from *C. gileadensis* leaf compared to the hydrodistillation extraction (HDE). Our results showed that the USE operating conditions were identified as: MeOH·H_2_O solvent‐to‐fresh sample ratio of 80:20 (v/v); ultrasonic power/frequency at 150 W/20 kHz; and a temperature of 40 ± 1°C; subjected to acoustic waves intermittently for a calculated time (5 min) during the total programmed time of 12 min. The USE exhibited (118.71 ± 0.009 mg GAE/g DM) more amounts of all phenols than HDE (101.47 ± 0.005 mg GAE/g DM), and antioxidant (77.78 ± 0.73%, 75.27 ± 0.59% scavenging inhibition of DPPH), respectively. Anti‐aging and Cytotoxicity activities were investigated. The results of biological evaluations showed that the crude extracts of *C. gileadensis* significantly extended the replicative lifespan of K6001 yeast. In addition, in vitro cytotoxicity against the HepG2 cell line showed significant anticancer activity, and approximately 100 μg/mL is required to decrease viability compared with that of the control. This study is proven for a larger scale to extract and isolate compounds of *C. gileadensis* for potential utilization in the pharmaceutical industry. In conclusion, advanced methods afford an extract with high activity in the biological properties of the extract.

## Graphical abstract

The USE operating conditions were identified as: MeOH·H_2_O solvent‐to‐fresh sample ratio of 80:20 (v/v); ultrasonic power/frequency at 150 W/20 kHz; and temperature of 40 ± 1°C; with subjected to acoustic waves intermittently for a calculated time (5 min) during the total programmed time of 12 min.

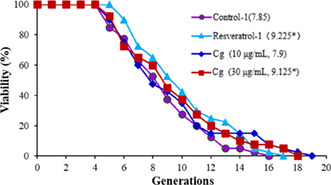

## 1 INTRODUCTION

Recently, various botanical extracts have gained special attention as natural sources of antioxidants and antimicrobiotic agents due to the potential toxicity of synthetic antioxidants and antibiotic resistance (Ielo et al., 2022). Phytochemicals such as phenolic compounds are a large group of substances and are found in large quantities in vegetables, fruits, spices, and seeds because they are considered potential antioxidant agents, and their role in the pharmaceutical and food industry and the chemical prevention of disease has become an area of active research in many areas (Maqsood et al., 2020). It has been shown that oxidative reactions are involved in aging and the development of many diseases. Therefore, it has been suggested that antioxidant molecules may slow the aging process, disease progression, and extend lifespan (Umaru et al., 2019).

Scientific research has focused on plant extracts as natural sources of antioxidant compounds (Kyzioł et al., 2021; Pirzadeh et al., 2021). Several studies have demonstrated the potential applications of medical, pharmaceutical, food industries, and cosmetics of secondary plant metabolites (Klimek‐szczykutowicz et al., 2020).

More than 60% of the overall time is spent on the sample preparation stage, so choosing the proper extraction method is very essential (Yahya et al., 2018). The extraction of bioactive compounds from plant sources can be improved with ultrasound technology assistance. The phenomenon of cavitation bubble collapse caused by acoustic and radiation shocks is responsible for the intensification of mass transfer, which facilitates the solvent to flow into plant tissues and thus increases the extraction efficiency of phenolic compounds (Kumar et al., 2021; Ojha et al., 2020).

Also, unlike conventional extraction methods like soaking and aqueous distillation, modern techniques can utilize the chemical and physical properties of the plant cell (such as osmotic pressure and water activity), which differ significantly when applied compared to conventional methods. Therefore, ultrasonic technology can increase the extraction yield, reduce chemical risks, and shorten the processing time (Švarc‐Gajić et al., 2013; Wang et al., 2012).

In the process of phenolic compound extraction, conventional extraction techniques such as maceration, percolation, and Soxhlet have been found to be extensively applied due to the simplicity of their operation and the simple equipment that is needed. Usually, extraction is carried out using organic solvents, which require a large volume of solvent and a long extraction period. In contrast, water is utilized as a solvent in the hydrodistillation and decoction methods. However, long extraction times, high amounts of organic solvents, and high energy consumption could lead to low yields and degradation of the compounds (Cannavacciuolo et al., 2023; Jha & Sit, 2022).

Recent studies have revealed that ultrasound‐assisted, supercritical fluid and microwave‐assisted extraction techniques are environmentally friendly extraction methods (Alahmad et al., 2023). Ultrasound‐assisted extraction is one of the simplest and most cost‐effective technologies available for improving extraction efficiency, decreasing extraction time, and enhancing overall effectiveness of processing (Usman et al., 2022). The process of ultrasound‐assisted extraction (USE) involves the formation of bubbles by a process called “acoustic cavitation,” which is facilitated by ultrasonic waves' ability to cause expansion and compression cycles (Soltani Firouz et al., 2022). Cavitation forces create a localized pressure that ruptures cell walls in the matrix when these bubbles burst, releasing their contents (Jadhav et al., 2023). Therefore, an ultrasound‐assisted technique may extract phenolic compounds quickly and easily.

Most plant sources, including those in the Burseraceae family, contain polyphenolic compounds or their derivatives. The Burseraceae family has promising potential for use as antioxidants and antimicrobials in the pharmaceutical and food industries (Afonso et al., 2018; da Silva et al., 2022; Emmanuelle Sika et al., 2021; Ifejirika et al., 2022).

Plant phenols are essential because they are low‐molecular‐mass antioxidants that can be obtained from food (Nadeem et al., 2022). In vitro and in vivo, several plant phenols demonstrated antibacterial or antioxidant activity (Prior, 2003). The intensity of the antioxidant activity depends on many factors, such as the number of hydroxyl groups attached to the aromatic ring (Mercado‐Mercado et al., 2020), and the number and locations of the double bonds in the molecule (Singla et al., 2019).

The objective of this study is to evaluate the antioxidant activity of three vital Yemeni plant species, including *Commiphora gileadensis*, *Indigofera spinosa*, and *Salvadora persica* (Table 1), and the total phenolic content (TPC) in the extracts of these plants by comparing the ultrasonic‐assisted technique with the hydrodistillation method to choose the best plant with the highest of total phenolic content and the highest antioxidant capacity for subsequent studies as study the anti‐aging activity and cytotoxicity to better contribute to the promotion of the use of natural compounds as an important and safe.

**TABLE 1:** Description of collected plant species investigated in this study.

Scientific name	Common name	Local name	Family
*C. gileadensis*	Balsam or bisham	Balsam	*Burseraceae*
*I. spinosa*	Hal	Hal	*Fabaceae*
*S. persica*	Arak, miswak	Arak	*Salvadoraceae*

## 2 MATERIALS AND METHODS

### 2.1 Plant sample

The aerial part (leaves) of *Commiphora gileadensis*, *Indigofera spinosa*, and *Salvadora persica* were collected during March–September 2021 from the Lawdar region (Village) in southwestern Yemen. It is located in Abyan Governorate, and the geographic coordinates are longitude (45°51′53″E) and latitude (13°53′11″N). This place is 3254 ft above sea level. Dr. Abdul Wali A. Al‐Khulaidi (Plant Geography, Flora and Vegetation, The Centre for Middle Eastern Plants [CMEP]) carried out the identification of botanicals. The leaves of each plant were dried at room temperature (23–25°C) for 10 days in a shaded place and ground using an electric grinder. The acceptable powder samples were kept in self‐sealing polyethylene plastic bags at 4°C.

### 2.2 Chemicals

Analytical‐grade chemicals and solvents were used in the experiments. Folin‐Ciocalteu (F–C) reagent and 3,4,5‐trihydroxybenzoic acid (gallic acid) were obtained from Sigma (Germany). di(phenyl)‐(2,4,6‐trinitrophenyl) iminoazanium (DPPH) and TBHQ were purchased from TCI EUROPE N.V. (Belgium). Glucose, galactose, agar, yeast, and peptone were purchased from Hangzhou Haotian Biotechnology Co., Ltd. (China). Hard‐Plus Resin‐812 (GP18010), uranyl acetate (GZ02616), and lead citrate (GZ02616) from Beijing Zhongjingkeyi Technology Co., Ltd (ZJKY; China). Osmium tetroxide (GP18456) from Leica (Germany). Alcohol (20191022) from Chengdu Kelon Chemical Reagent Factory (China). PBS Buffer Powder (YM‐XZ002) from Shanghai Yuanmu Biotech (China).

### 2.3 Extraction procedures

Two extraction techniques were employed for hydrodistillation extraction (HDE) and ultrasonic‐assisted extraction (USE).

Equation (1) was used to determine the extraction yield (%):

(1) $$\text{Yield}\%=\left(\text{Weight of dried extract}/\text{Weight of sample}\right)\times 100$$

#### 2.3.1 Hydrodistillation extraction (HDE)

To study the antioxidant activities and TPC of the three selected medicinal plants, we used hydrodistillation extraction (HDE) as a conventional method to select the plants with high antioxidant and TPC. Upson et al. (2000) described the procedure followed with minor adjustments. Briefly, hydrodistillation was performed in a Clevenger‐type apparatus. For each plant sample, 4 g was added in a 500 mL hydrodistillation apparatus flask and dissolved in 100 mL of MeOH·H_2_O at a ratio of 80:20 (v/v). All prepared samples were heated for 60 min at 60°C. The supernatants were centrifuged for 10 min at 5000 rpm and 4°C after being filtered using Whatman paper No. 1. Also, filtering was repeated using a Buchner funnel and Whatman No. 1 paper under a vacuum. The volume was filled up to 100 mL with deionized distilled water and kept at 4°C until the next analysis.

#### 2.3.2 Ultrasonic‐assisted extraction (USE)

The ultrasonic power/frequency at 150 W/20 kHz was tuned for the USE operating conditions. In a coated glass container, with magnetic stirring, 100 mL of MeOH·H_2_O at a ratio of 80:20 (v/v) was used to dissolve 4 g of plant powder. The total programmed time was 12 min, and the mixture was treated to sonic waves intermittently for a considered time of 5 min. The temperature was maintained constant at 40 ± 1°C using a thermostatic external cold‐water bath. After that, filtration and solvent removal were carried out as shown in item (Section 2.3.1).

### 2.4 Analysis of *Cg*‐polyphenol extracts

#### 2.4.1 Determination of total phenolic content (TPC)

**FIGURE 1:**
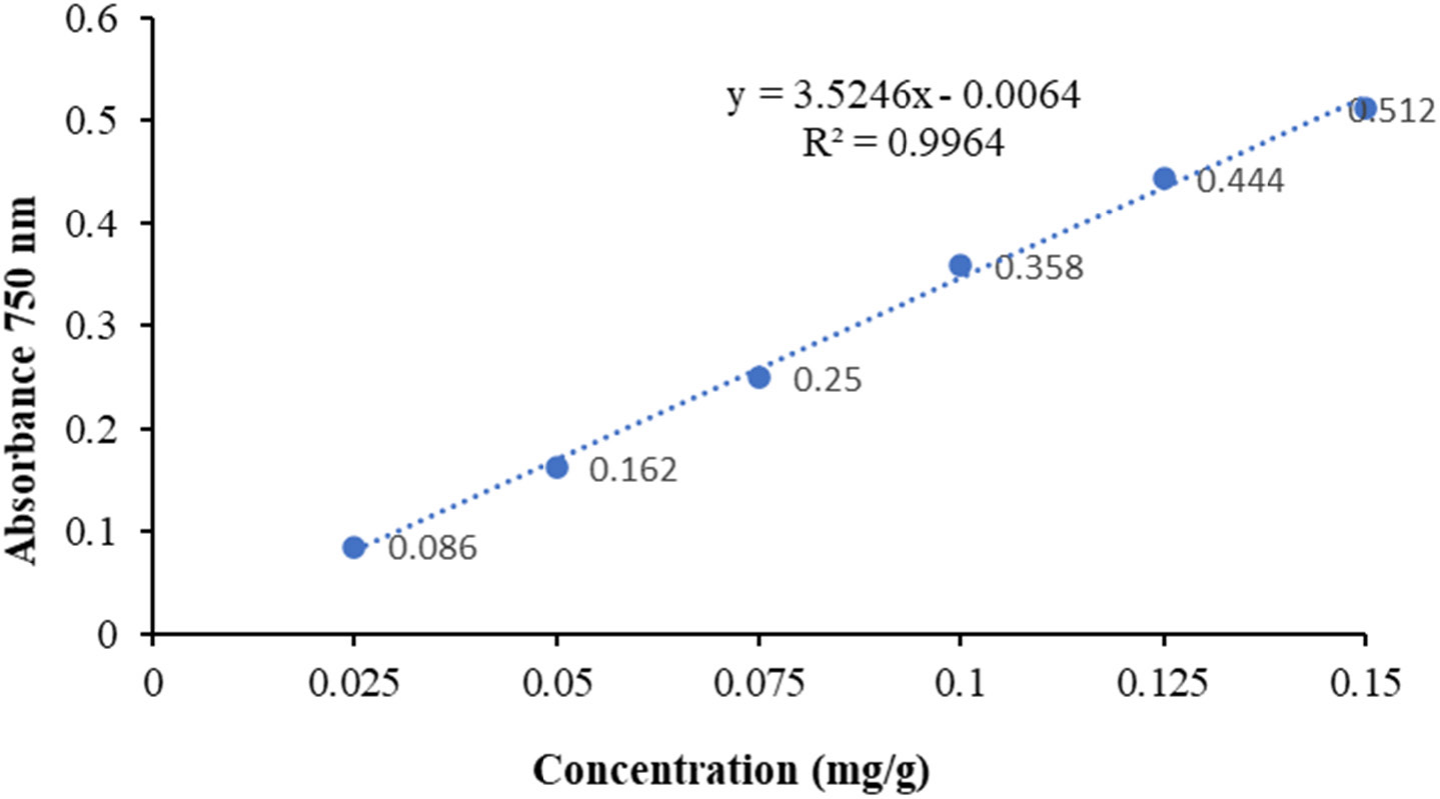
Calibration curve for gallic acid (mg/g of dry extract) for three plants.

As Rashed et al. (2016) described, Singleton and Rossi's colorimetric oxidation reaction technique was used to determine the TPC of plant powder extract. The results were presented as mg gallic acid equivalents per gram of dry matter (mg GAE/g DM), using the standard curve of gallic acid (*R* ^2^ = .99; Figure 1).

#### 2.4.2 Determination of antioxidant activity using the DPPH

The antioxidant scavenging effect of extracts was assessed based on the DPPH assay (Rashed et al., 2016), according to Rashed et al. without modification. The results were expressed according to the following equation:

(2) $$\text{DPPH}\%=\left[\left(A-B\right)/A\right]\times 100$$

where *A* is the optical density of the control sample and *B* is the optical density of the sample. TBHQ served as a positive model at 120 mg/L. There were three duplicates of each treatment.

#### 2.4.3 Lifespan assay of *C. gileadensis*

Based on Weng et al. (2010), the bioassay method was used to study the anti‐aging activity. A yeast strain (K6001) was grown in YPGalactose or YPGlucose mediums. Galactose (3%), hipolypeptone (2%), and yeast extract (1%) were used to prepare YPGalactoses, while glucose was used to prepare YPGlucoses, which included 2% glucose instead of galactose. Agar plates were prepared by adding agar to the medium at a 2% concentration. To carry out bioassays, the yeast peptone galactose medium was used to cultivate the K6001 yeast strain for 24 h with shaking. After 24 h, centrifugation was performed on the yeast strain medium. The phosphate buffer solution (PBS) was used three times to rinse the diluted yeast pellets. Around 4000 cells were injected into agar plates containing various substances after the cells had been diluted. The plates were kept at 28°C in an incubator for the next 2 days. After 48 h, the agar plate containing the yeast cells was examined under a microscope. Each of the 40 microcolonies randomly selected from each plate was then analyzed and counted the number of daughter cells.

#### 2.4.4 Cell viability assay of *C. gileadensis*

Measurement of cell viability was determined using the HepG2 cells. In the assay for cell viability, 100 mL of DMEM was added to 96‐well plates, where 1 × 10^4^ cells per well were cultured overnight. *C. gileadensis* extract was added to cells in different dilutions (1, 10, and 100 μg/mL), and cultured at 37°C for 24 h with 5% CO_2_. Each well was incubated for 1 h at 37°C with 10 μL of CCK‐8 reagent before readings were taken at 450 nm with an optical microplate reader (Enspire2300).

### 2.5 Statistical analysis

All results were reported as a mean ± standard deviation after three replicates of each test. The SPSS 22.0 (SPSS Inc.) software program on Microsoft Windows 10 was used to conduct one‐way analysis of variance (ANOVA) utilizing Duncan's new multiple‐range test to determine statistically significant differences between means.

## 3 RESULTS AND DISCUSSION

### 3.1 Extraction yield (%)

Extract yield of *C. gileadensis*, *I. spinosa*, and *S. persica* leaves was prepared by hydrodistillation extraction and ultrasonic‐assisted extraction methods. The extraction yield (mass of extract/dry matter) was employed as an index of the extraction methods' effects. Based on the product obtained after evaporation, the yield percentage of the extracts of the three Yemeni medicinal plants was determined (Table 2; Akkol et al., 2011).

**TABLE 2:** Percentage yield of various extracts of three Yemeni medicinal plants.

Botanical name	Yield			
	HDE		USE	
	DM	%	DM	%
*C. gileadensis*	0.81 ± 0.02	20.25	0.44 ± 0.05	11.00
*I. spinosa*	0.63 ± 0.03	15.75	0.27 ± 0.03	6.75
*S. persica*	0.73 ± 0.04	18.25	0.25 ± 0.02	6.25

Abbreviations: DM, dry matter; HDE, hydrodistillation extraction; USE, ultrasonic‐assisted extraction.

The quantitative estimation of the percentage yield of various crude extracts of the plants is calculated as the weight (g) of crude extracts obtained from 4 g dried leaves. The results of the percentage yield are adequately described by Equation (1).

Among the hydrodistillation extracts of all three plants, the yield of *C. gileadensis* (20.25%) showed the highest yield percentage, and the lowest yield percentage was observed in the extract of *I. spinosa* (15.75%) among the three plants studied by hydrodistillation extraction. The yield of *S. persica* was 18.25%. While the ultrasonic extracts of *C. gileadensis* (11.00%) showed the highest yield percentage, the lowest yield percentage was observed in the extract of *S. persica* (6. 25%) among the three plants studied by ultrasonic‐assisted extraction. The yield of *I. spinosa* was 6.75% (Table 2).

The extract yield of *I. spinosa* and *S. persica* with the conventional method was about 2.6 times higher than that of an advanced method for the same plant. This could be due to the more vital polarity of water, as well as the fact that at elevated extraction temperatures, and in the case of HDE, water has a similar dielectric constant to organic solvents such as methanol and acetonitrile. In the case of HDE, the extract yield increased with the time of extraction by the conventional method. The extract yield of *C. gileadensis* by ultrasonic‐assisted method was lower than the maximum extract yield obtained by the conventional method may be due to the time of extraction.

### 3.2 Effect on TPC of three plants

Polyphenols have a vital role in fruit and vegetable color and flavor and protect from oxidation in vivo and in vitro. Antioxidants in the phenolic class transfer one electron to free radicals, making them more stable by pairing their electrons. This protects cells and tissues from oxidative stress‐induced damage (Andzi Barhé & Feuya Tchouya, 2016). The phenolic content is considered an important indicator of antioxidant capacity.

Many researchers have investigated the best methods for extracting plant‐based bioactive compounds such as polyphenols and phenolic acids. Several evaluations have been documented, including the principles of these approaches and the factors that influence them, as well as a comparative analysis of their respective merits (Uwineza & Waśkiewicz, 2020). Conventional extraction methods of extraction are still used despite the significant drawbacks, such as long extraction times and the large amounts of organic solvents used.

In recent years, the demand for modern extraction technologies that are ecologically friendly, more efficient, and faster than conventional methods has increased. Many studies have compared modern techniques for extracting medicinal plants, either among themselves or with conventional methods (Jovanovic et al., 2017). Although modern techniques offer several benefits for extracting biomolecules from various plants, notably concerning extraction time, solvent consumption, and extraction yields, the best methods for extracting bioactive compounds need to be carefully evaluated (Fierascu et al., 2020; Herrero et al., 2015).

In this work, the TPC of the dried leaves of three plants (*C. gileadensis*, *I. spinosa*, and *S. persica*) in Yemeni's system of medicine has been investigated and determined as shown in Table 3. The total phenolic contents were also extracted by ultrasonic‐assisted technique as an advanced technique compared to the conventional hydrodistillation method. The antioxidant activity of both the ultrasonic‐assisted extraction and conventional hydrodistillation methods was determined for the three plants selected in this study.

**TABLE 3:** Total phenolic content and inhibition of DPPH for three medicinal plants prepared using different extraction methods.

Botanical name	Phenolic content (TPC; mg GAE/g DM)		TPC increase rate (%)	DPPH scavenging Inhibition of DPPH (%) [100 μg/mL]		DPPH (% inhibition) Increase rate (%)
	HDE	USE		HDE	USE	
*C. gileadensis*	101.47 ± 0.005	118.71 ± 0.009	16.9	75.27 ± 0.59	77.78 ± 0.73	3.3
*I. spinosa*	38.22 ± 0.007	51.86 ± 0.010	35.9	55.08 ± 0.73	60.05 ± 0.30	9.1
*S. persica*	13.23 ± 0.006	18.33 ± 0.008	38.6	12.34 ± 0.79	16.88 ± 0.71	37.4
TBHQ	ND			89.74 ± 0.46		

*Note*: Means of three determinations ± standard deviation; the percentage increases in TPC and DPPH (% inhibition) between the three plants are shown in separate columns.

Abbreviations: GAE, gallic acid equivalent; HDE, hydrodistillation extraction; ND, not determined; USE, ultrasonic‐assisted extraction.

The total phenolic content by hydrodistillation extraction was (101.47 ± 0.005, 38.22 ± 0.007, and 13.23 ± 0.006) mg/g extract in *C. gileadensis*, *I. spinosa*, and *S. persica*, respectively. The results indicated significant differences among extracts, where the plant extracts of *C. gileadensis* contained higher phenol content than other extracts. In contrast, the ultrasonic extracts of the total phenolic content were (118.71 ± 0.009, 51.86 ± 0.01, and 18.33 ± 0.008) mg/g extract in *C. gileadensis*, *I. spinosa*, and *S. persica*, respectively.

The effectiveness of extraction from all three of the medicinal plants under investigation was enhanced by ultrasonic power. When using the ultrasonic extraction method, it was noted that it is accompanied by an increase in the total phenolic content of all plants in varying proportions (Table 3). The highest percentage increase in TPC has been in the *S. persica*, where the amount of extraction in the conventional method was 13.23 mg GAE/g DM, while the amount increased using the ultrasonic‐assisted method to 18.33 mg GAE/g DM, and the percentage of increase reached 38.6%. On the opposite, the lowest percentage increase was in the *C. gileadensis*, where the yield extraction value in the conventional method was 101.47 ± 0.005 mg GAE/g DM. The amount was increased after sonication treatment to 118.71 ± 0.009 mg GAE/g DM, and the percentage of increase reached 16.9%.

As well as a rise in TPC, an increase in solubility of solute and solvent diffusivity may have resulted from an increase in temperature. Due to the sonication‐generated acoustic cavitation bubbles increasing the extraction temperature and destroying the plant cell wall, more phenolic compounds were released into the solution matrix (Altemimi et al., 2016).

The process of extraction is the major difference between ultrasonic extraction and hydrodistillation. The ultrasonic extraction method utilizes high‐frequency sound waves to generate cavitation in the solvent, which helps break down the plant material's cell walls to extract the bioactive compounds. This is due to ultrasonic extraction's ability to extract volatile and non‐volatile compounds from the plant material (Dash et al., 2021). TPC extraction from *C. gileadensis* leaf extract using ultrasonic‐assisted extraction (UAE) is superior to the hydrodistillation method. Based on the results of this investigation, USE is a potential approach for the extraction of bioactive compounds from *C. gileadensis* since it has various benefits over conventional extraction methods. Using ultrasound waves enhances the extraction process's efficiency and releases bioactive compounds from *C. gileadensis* leaf. Our result is consistent with the Qiu et al. study, which demonstrated that ultrasound enhances the efficiency of the extraction process by disrupting cell walls and releasing bioactive compounds (Qiu et al., 2022). In contrast to HDE, which often requires high temperatures and thus can cause the thermal degradation of heat‐sensitive compounds, the USE technique only needs lower extraction temperatures. It thus preserves the bioactivity of the compounds (Mehmood et al., 2019). With increased solubility, ultrasound can also help to increase the solubility of compounds that are otherwise difficult to extract using the HD method (Medina‐Torres et al., 2017). The efficacy of ultrasonic‐assisted extraction is shown through the improved bioactive compound yield and helps reduce the extraction time. Ultrasonic waves are capable of making physical alterations on solid surfaces. This could be one of the reasons for the reduction in extraction time (Jain et al., 2022). The maximum possible valued phenolics might be extracted in 12 min as phenolic contents are commonly observed at the epidermis region of leaves. Due to sonication, they could have been released faster. This study revealed that the ultrasonic‐assisted conditions of the three extraction steps of 12 min, 40°C, and MeOH·H_2_O were more efficient in comparison to the conventional extraction.

**FIGURE 2:**
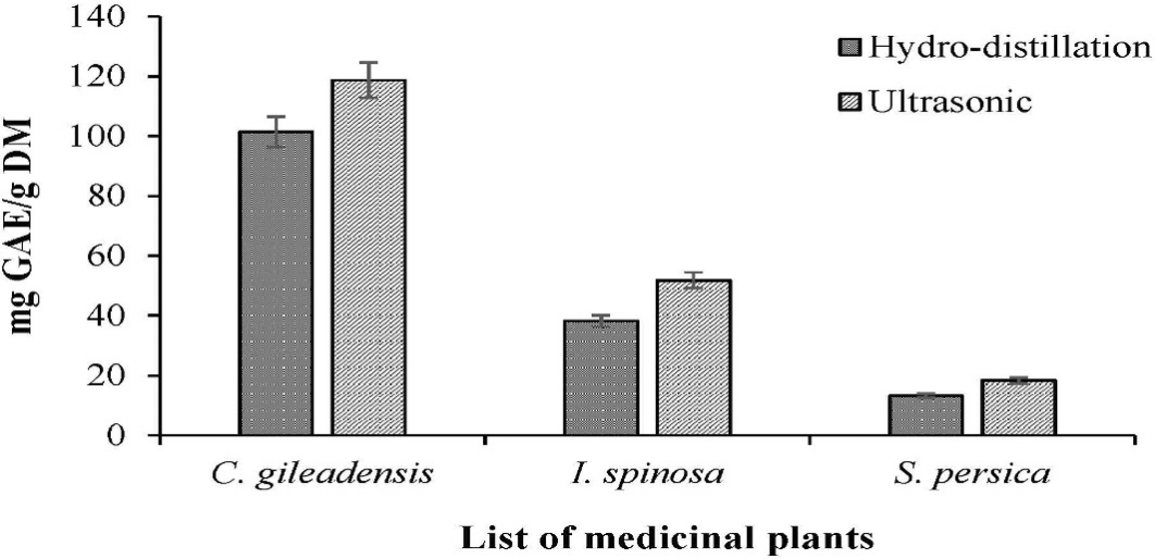
Comparison of TPC obtained by HDE and USE.

The total phenolic content of the three medicinal plants varied among the analyzed varieties (Figure 2). The total phenolic variation in this study may have been caused by the different samples, geographic locations of samples, and cultivars studied.

### 3.3 Antioxidant activity using (DPPH) radical scavenging

Antioxidants are substances that can scavenge free radicals and prevent the oxidative damage they generate in cells. Lowering oxidative damage is very important for preventing cancer and other diseases (Mfotie Njoya, 2021).

In comparison to *C. gileadensis*, *I. spinosa*, and *S. persica*, extracts of *C. gileadensis* had stronger scavenging values, when tested at a concentration of 100 μg/mL, the extracts of *C. gileadensis* had antioxidant activity of 77.8%, while the extracts of the other two plants were less effective. One explanation for these results is that radical‐scavenging capacity depends not just on the redox potentials of compounds but also on their hydrogen‐donating ability (Lucarini et al., 1999). The high polyphenol content may be a contributing factor. Other investigations have found a similar link between antioxidant activity and phenolic concentration (Apak et al., 2007; Ricci et al., 2006).

Many functional groups, such as hydroxyl and carbonyl groups, have been linked to antioxidant activity in these extracts (Galati & O'Brien, 2004; Payet et al., 2005). The DPPH assay revealed that several extracts exhibited high levels of antioxidant activity. The efficiency of antioxidants is mostly dependent on the concentrations of the extracts. Their ability to free radical scavenger was ranked in the following ascending order: *S. persica* ˂ *I. spinosa* ˂ *C. gileadensis*.

**FIGURE 3:**
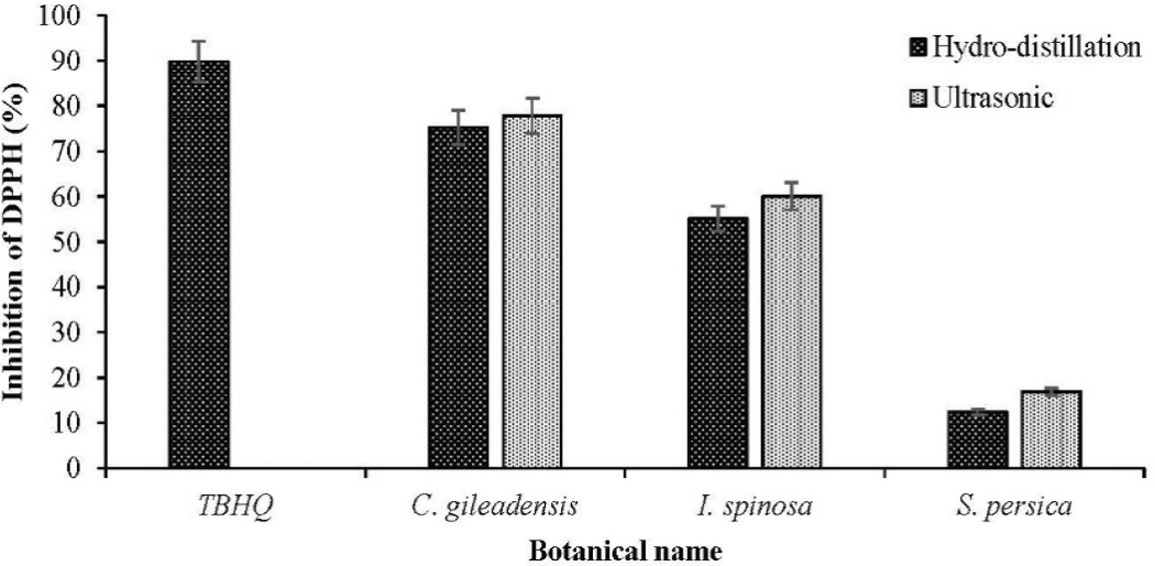
Comparison of DPPH obtained by HDE with USE.

Hydrodistillation extracts showed that extracts of *C. gileadensis* were fairly close inhibitors of DPPH scavenging to reference antioxidants such as TBHQ (Table 3). The inhibition of DPPH % of *C. gileadensis* was 75.2%, indicating that their DPPH scavenging antioxidant activity is less than extraction by USE method by about 3.3%. The extracts of *C. gileadensis* exhibited stronger DPPH scavenging activities compared to other plants, possibly due to their extract's components, such as tocopherols, phytosterols, and phenolic compounds. The DPPH of *C. gileadensis* has a stronger antioxidant activity when compared to *S. persica*, and *I. spinosa* (Figure 3) by both extraction methods. To compare DPPH scavenging activities of the USE and HDE antioxidant activity values, *C. gileadensis* showed significantly higher antioxidant activity than *S. persica* and *I. spinosa*.

Ultrasonic‐assisted extraction has been shown to be an effective method for improving the yield of antioxidants from *C. gileadensis* compared to the HDE method. This is because the ultrasonic energy helps break down the plant material's cell walls, which increases the availability of antioxidants for extraction (Mounika et al., 2022). However, the amount of antioxidants obtained through ultrasonic‐assisted extraction depends on extraction parameters such as ultrasonic energy duration, frequency, and intensity, as well as the type and amount of solvent used. (More & Arya, 2021). Extracting antioxidants from *C. gileadensis* using ultrasound‐assisted extraction is a promising approach. Although the antioxidant yield of *C. gileadensis* leaves was increased through the use of ultrasound‐assisted extraction, the mechanism may involve a combination of physical and chemical effects, including cell wall disruption, solvent permeation, temperature control, and enhancement of chemical reactions. These effects enhance the mass transfer of the antioxidants from the plant material into the solvent, leading to a greater yield of antioxidants compared to HDE method (Dash et al., 2021; Khadhraoui et al., 2021).

### 3.4 Anti‐aging effect

Aging is a biological fact and an inevitable process that has its way of occurring beyond human control, which increases the prevalence of age‐related illnesses.

The majority of anti‐aging agents are derived from plants, such as resveratrol. The K6001 yeast strain was used to build a convenient bioassay system to evaluate the anti‐aging activity of natural product extracts. The anti‐aging activity of the samples was investigated by counting the number of daughter cells produced before the death of yeast mother cells. The data analysis was done through the statistical software of SPSS, 40 data of each sample were analyzed, the average yeast life of each group was calculated, and a one‐to‐one *T*‐test was carried out between each group and the negative control group. The *p*‐value determined the active size of the sample. Resveratrol was employed as the positive control group, while untreated was used as the negative control group.

**FIGURE 4:**
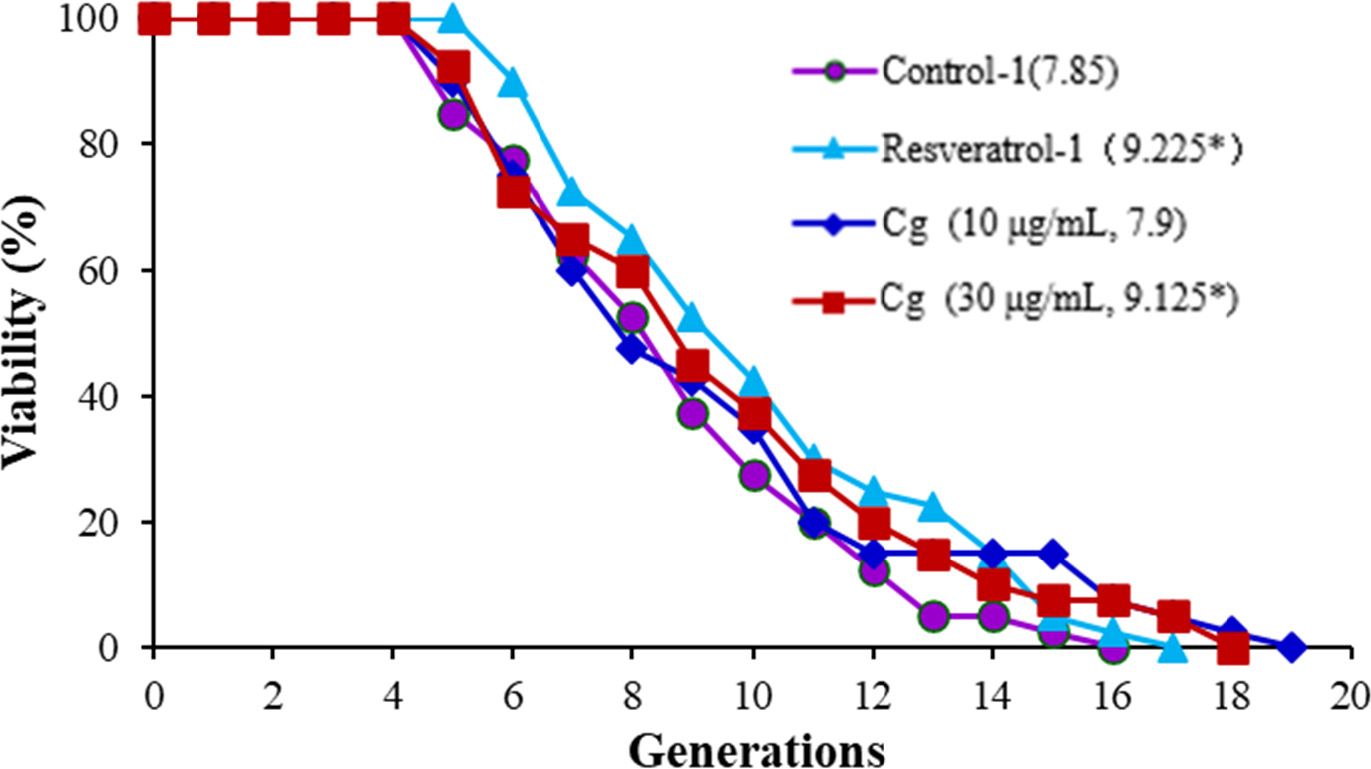
Effect of *C. gileadensis* (*Cg*) on the replicative lifespan of the K6001 yeast strain. Average lifespan of K6001 was as follows: control (7.85 ± 0.34); resveratrol at 10 μg/mL (9.225 ± 0.53*); and *Cg* at 10 μg/mL (7.9 ± 0.36) and at 30 μg/mL (9.125 ± 0.62*) (*p < .05, compared with the control).

As shown in Figure 4, the result revealed that *C. gileadensis* compared with the negative control, the replicative lifetime of the K6001 yeast strain increased significantly. The average lifespan of the negative control was 7.55 ± 0.48. In comparison, the K6001 at 30 μg/mL of *C. gileadensis* (9.15 ± 0.62) increased significantly, whereas the *C. gileadensis* is inactive at 10 μg/mL (8.025 ± 0.41) as shown in Figure 4. The mentioned finding suggested that *C. gileadensis* had anti‐aging properties in yeast.

Based on previous studies of *C. gileadensis* and thorough examinations of the chemical components of the leaves (Dudai et al., 2017), the analysis showed the abundance of monoterpene hydrocarbons. α‐Pinene (18.4%), β‐caryophyllene (20.12%), β‐pinene, terpinen‐4‐ol, and sabinene were the most plentiful components in the essential oils of *C. gileadensis* leaves. These could constitute a consolidated source of natural bioactive ingredients with anti‐aging and antimicrobial properties. According to Gerofotis et al. (2016), olive fruit flies (*Bactrocera oleae*) exposed to the aroma of α‐pinene, which is found in olive fruit and leaves, under dietary restriction, the lifespan in males and fecundity in females increased. Besides, only β‐caryophyllene, a compound with a wide range of biological effects, was the subject of lifetime extension studies. Pant et al. (2014) employed *C. elegans* as a model system to clarify how β‐caryophyllene reduces stress modulatory and lifespan‐extending properties. The results were shown to increase the *C. elegans* lifespan by more than 22% with a 50 M dosage of the sesquiterpene. Furthermore, the levels of intracellular free radicals were significantly reduced, and the maintenance of cellular redox equilibrium, while the primary anti‐aging genes SIR‐2.1, SKN‐1, and DAF‐16 in *C. elegans* interact with β‐caryophyllene (Pant et al., 2014). Most classes of terpenoids have the potential to increase the average lifespan, and non‐identified compounds of *C. gileadensis* leaves probably have a substantial contribution to the bioactivities. These compounds have a great prospect of becoming a new class of anti‐aging drugs.

### 3.5 In vitro cytotoxicity

In 2018, liver cancer had the third‐highest cancer‐related deaths; by 2020, it had increased to the second‐highest mortality rate worldwide (Cao et al., 2021). Natural products are one of the important alternatives for drugs against cancers, including liver cancer. Many phenolic chemical prevention studies focus on liver cell response, as the liver is the primary site for xenobiotic metabolism (Arora et al., 2019; Nardo et al., 2021). The human hepatoma cell line (HepG2 cells) is a good model for studying the xenobiotic metabolism of microorganisms in vitro and liver toxicity because they maintain many specialized functions characteristic of normal human hepatocytes (Štampar et al., 2019). The HepG2 model has also been widely used in biochemical and nutritional studies where many compounds and conditions have been tested using minor intravariability (Ding et al., 2019).

**FIGURE 5:**
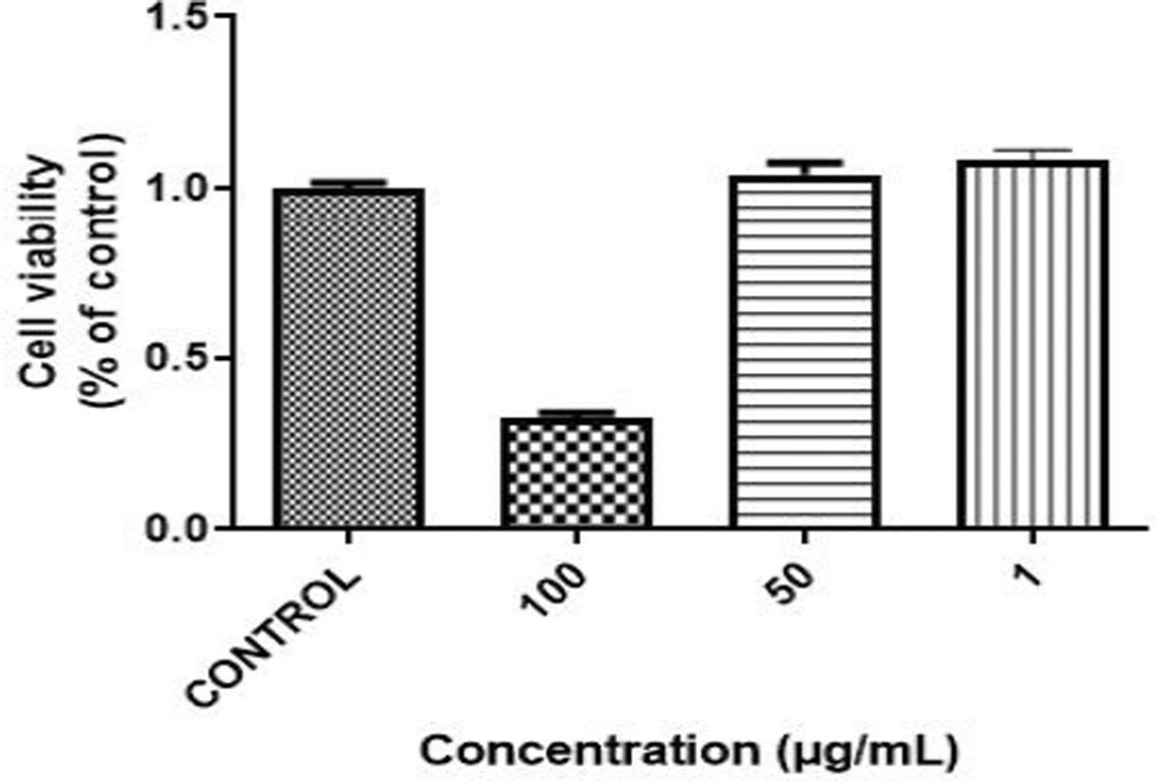
Anticancer activity of various concentrations of *C. gileadensis* against the liver cancer cell line—HepG2.

The CCK‐8 assay was used to investigate in vitro cytotoxicity against the HepG2 cell line at various concentrations of *C. gileadensis* extract (1, 50, and 100 μg/mL). After 24 h of treatment, the cell viability was assessed. Figure 5 shows the substantial anticancer effect of the *C. gileadensis* extract, with a concentration of 100 μg/mL required to reduce viability compared with that of the control, but *C. gileadensis* extract showed inactive cytotoxicity in concentrations of 1 and 50 μg/mL. In contrast, 100 μg/mL *C. gileadensis* extract significantly inhibited cell growth. The anticancer activity is directly dose dependent, demonstrating the critical importance of the *C. gileadensis* concentration as cell viability decreased with the increase in the extract concentration in a dose‐dependent manner.

Our results collaborated with earlier observations of the effect of *C. gileadensis* extracts on the anticancer and hepatoprotective (Al‐Zahrani et al., 2022; Doa rsquo a et al., 2016).

In previous studies, aliphatic alcohol glycosides, triterpenoids, sesquiterpenoids, and several new cycloartane types were isolated from *C. gileadensis*, and the cytotoxic effect of *C. gileadensis*, especially against human prostate, liver, and cervical cancer cell lines, was attributed to these phytochemicals (Al‐Zahrani et al., 2022; Blowman et al., 2018). β‐caryophyllene is an active component in the ethanolic extracts of *C. gileadensis* and inhibits growth and induces apoptosis in two lymphocytic tumor cell lines (Wineman et al., 2015). Several therapeutic uses of *C. gileadensis* have been reported and include the antiproliferative properties of the ethanolic extract of *C. gileadensis* sap in cancer cell lines, especially in strongly inducing apoptosis in immortalized and transformed human epidermal cell lines (Al‐Zahrani et al., 2022; Wineman et al., 2015), therefore, required to identify the possible mechanism involved in the anticancer activity.

## 4 CONCLUSION

In sum, following the use of different extraction methods, the ultrasonic‐assisted extraction employing an ultrasonic device with direct agitation resulted in the highest polyphenols with higher antioxidant activity compared to conventional extraction. Direct sonication with a probe system was more efficient than conventional extraction. The values of total phenol extracted with ultrasonic‐assisted extraction under the following conditions are as follows: MeOH·H_2_O solvent‐to‐fresh sample ratio of 80:20 (v/v); ultrasonic power/frequency at 150 W/20 kHz; and the temperature of 40 ± 1°C; with subjected to acoustic waves intermittently for a calculated time (5 min) during the total programmed time of 12 min; were higher than conventional extraction (60°C, 60 min) with a significant shortening of processing time. Furthermore, based on the findings of this study, *C. gileadensis* demonstrated promising bioactivity. They can thus be considered a potential plant‐based polyphenol agent for anti–free radicals, anti‐aging, and HepG2 protection. Further studies using more technical methods to elucidate the exact constituent (s) responsible for these benefits without side effects are required in order to approve and expand these findings.

## CONFLICT OF INTEREST STATEMENT

The authors declare no conflict of interest.

## ETHICS STATEMENT

This article does not involve humans or animals.

## CONSENT TO PARTICIPATE

All the co‐authors participated in the preparation of this manuscript.

## Data Availability

The data that support the findings of this study are available on request from the corresponding author.
